# Characterization of Biosynthetic Genes of Ascamycin/Dealanylascamycin Featuring a 5′-O-Sulfonamide Moiety in *Streptomyces sp.* JCM9888

**DOI:** 10.1371/journal.pone.0114722

**Published:** 2014-12-05

**Authors:** Chunhua Zhao, Jianzhao Qi, Weixing Tao, Lei He, Wei Xu, Jason Chan, Zixin Deng

**Affiliations:** 1 Key Laboratory of Combinatory Biosynthesis and Drug Discovery (Ministry of Education) and School of Pharmaceutical Sciences, Wuhan University, 185 East Lake Road, Wuhan, 430071, PR China; 2 Department of Chemistry, The Hong Kong University of Science and Technology, Clear Water Bay, Kowloon, Hong Kong; Woosuk University, Republic Of Korea

## Abstract

Ascamycin (ACM) and dealanylascamycin (DACM) are nucleoside antibiotics elaborated by *Streptomyces sp*. JCM9888. The later shows broad spectrum inhibition activity to various gram-positive and gram-negative bacteria, eukaryotic *Trypanosoma* and is also toxic to mice, while ascamycin is active against very limited microorganisms, such as *Xanthomonas*. Both compounds share an unusual 5′-*O*-sulfonamide moiety which is attached to an adenosine nucleoside. In this paper, we first report on the 30 kb gene cluster (23 genes, *acmA* to *acmW*) involved in the biosynthesis of these two antibiotics and a biosynthetic assembly line was proposed. Of them, six genes (AcmABGKIW) are hypothetical genes involved in 5′-O-sulfonamide formation. Two flavin adenine dinucleotide (FAD)-dependent chlorinase genes *acmX* and *acmY* were characterized which are significantly remote from *acmA-W* and postulated to be required for adenine C2-halogenation. Notably gene disruption of *acmE* resulted in a mutant which could only produce dealanylascamycin but was blocked in its ability to biosynthesize ascamycin, revealing its key role of conversion of dealanylascamycin to ascamycin.

## Introduction

A number of nucleoside antibiotics have been discovered in the past half century and some of them were demonstrated to be particularly active antibiotics, pesticides and fungicides which have been developed into agrochemicals or pharmaceuticals [1]. Examples of nucleoside antibiotics include puromycin [2], nucleocidin [3], toyocamycin and sangivamycin [4], polyoxin [5], pacidamycin [6] [7], blasticidin S [8], nikkomycin [9] [10], gougerotin [11], A-500359s and A-503803s [12], caprazamycin [13], liposidomycin and A-90289 [14], muraymycin [15], mildiomycin [16], tunicamycin [15] [17] and amicetin [18]. Most of the biosynthetic pathways for the above nucleoside compounds have been reported in the past decade, which paved the way to the characterization of new enzymes and provided useful insights in devising novel antimicrobial agents [19].

**Figure 1:**
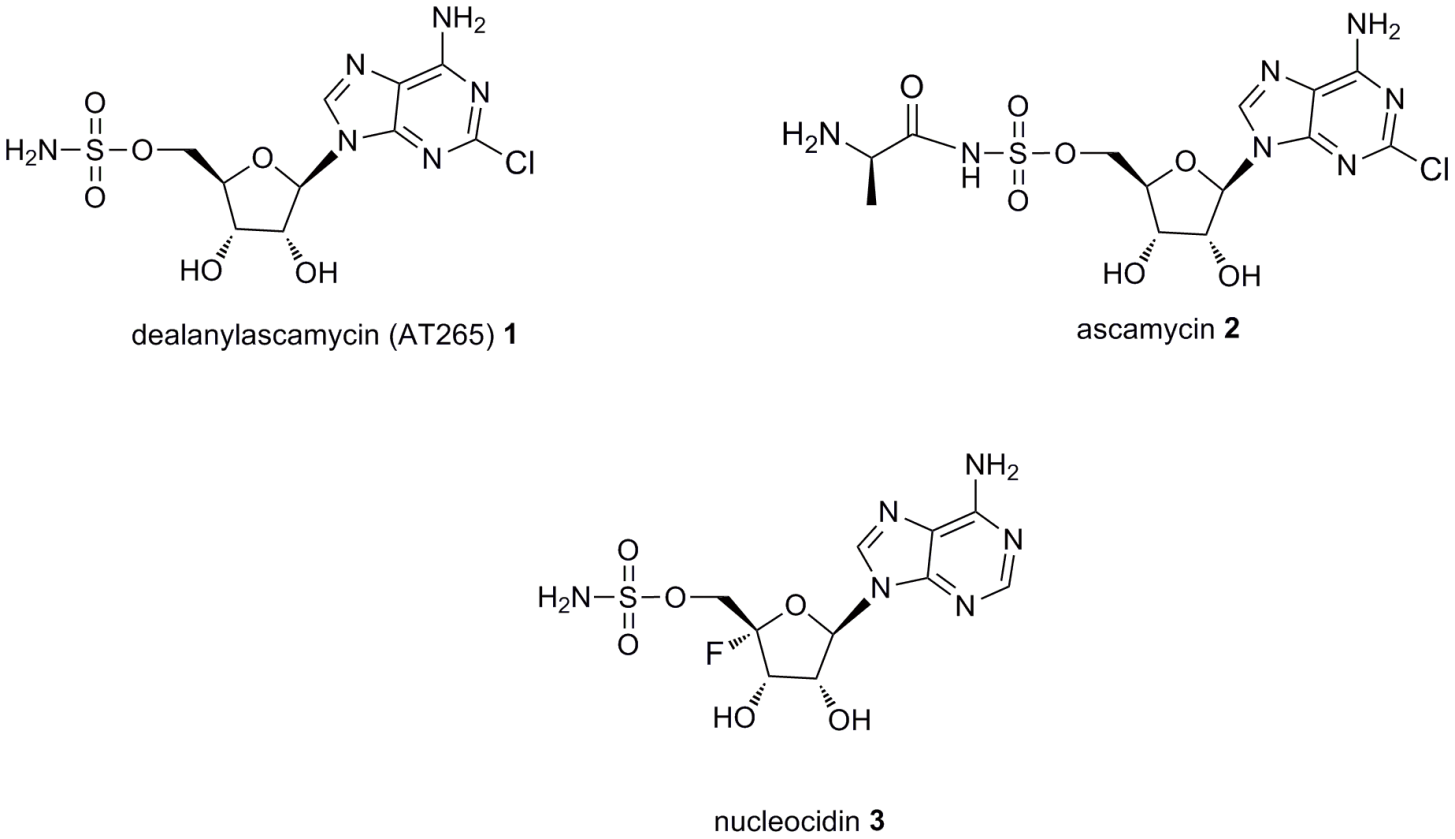
Chemical structures of nucleoside antibiotics in this study: dealanylascamycin 1, ascamycin 2, nucleocidin 3.

Dealanylascamycin (DACM) (**1**) and ascamycin (ACM) (**2**) [20] (also identified as AT-265 [21]) (Fig. 1) are two adenosine antibiotics produced by *Streptomyces sp.* JCM9888 whose chemical scaffold resemble that of the fluorine-containing antibiotic nucleocidin (**3**) [3] (Fig. 1). Ascamycin, dealanylascamycin and nucleocidin are closely related 5′-O-sulfonamide ribonucleosides. The ascamycins have C2-chloroadenine as the base on C-1′, whereas nucleocidin uses adenine, which lacks the chlorine. Nucleocidin also has a unique fluorine atom on its C-4′ position. Ascamycin and dealanylascamycin differ by *N*-alanylation of the 5′-O-sulfonamide moiety in the former but not the latter (Fig. 1) [3]. Although ascamycin and dealanylascamycin have similar chemical structures, they possess very different bactericidal activities. Ascamycin exhibited toxicity only to a few bacterium genus, such as *Xanthomonas* [22], while dealanylascamycin is broad-spectrum and active against both gram-positive, gram-negative bacteria and even some eukaryotic cells [22]. The later also showed unusual trypanocidal (such as *Trypanosoma equiperdum*) activity and is an anti-amoebae (such as *Endamoeba histolytica*) agent and thus it has been found valuable in treating animal diseases caused by these microorganisms [23]. Their differences in biological activity has been attributed to an aminopeptidase that cleaves the alanyl group from ascamycin. Since ascamycin is only active against a few microorganisms (*Xanthomonas*) in which this aminopeptidase was found, it was proposed that dealanylascamycin is the active form of this antibiotic, and most microorganisms (such as *E. coli* etc.) lack this aminopeptidase and are therefore not susceptible to ascamycin [22], [24]. Ascamycin and dealanylascamycin both possess a chlorine atom on the C2 position of adenine. It is often the case that incorporation of halogen into natural products plays an important role in increasing their biological scope and activity [25]. Some naturally occurring halogenated secondary metabolites, such as vancomycin, chlorotetracycline are of clinical importance. The mechanisms of biohalogenation, particularly for chlorination and bromination were widely discussed [25] [26] [27]. Enzymes involved in halogenation of aromatic groups have been extensively described, which require a two-component flavin-dependent halogenase/reductase as follows: A flavin reductase uses NADH (Nicotinamide adenine dinucleotide) to reduce FAD (flavin adenine dinucleotide) and produces FADH_2_ (reduced flavin adenine dinucleotide) and a halogenase utilize the FADH_2_ formed and O_2_ as cofactors to perform electrophilic halogenation *via* an intermediate halonium ion equivalent [25]. The mechanism of dealanylascamycin inhibition has been studied in the analogous antibiotic nucleocidin, which demonstrated that it inhibits amino acid incorporation into human liver cell protein *in vivo* and *in vitro* [28]. Due to their special biological activities, organic chemists have been attracted to synthesize ascamycin, dealanylascamycin, nucleocidin and related nucleoside derivatives [29] [30] [31].

In this paper we first report the entire gene cluster (*acmA*-*acmW*) responsible for the biosynthesis of two nucleoside antibiotics ascamycin/dealanylascamycin and a biosynthetic machinery was purposed from *in silico* analysis. Intriguingly, six genes (AcmABGKIW) are postulated to be involved in 5′-O-sulfonamide formation. Two FADH_2_-dependent halogenases (*acmX* and *acmY*) which are involved in the pathway were found not clustered with *acmA-acmW* and we postulate that they are required for chlorination on the C2-position of the adenine ring. Notably, inactivation of an esterase *acmE* resulted in a mutant which only produce dealanylascamycin but blocked in its ability to biosynthesize ascamycin which suggest its role of converting dealanylascamycin to ascamycin. These results provide biosynthetic profiles for the 5′-O-sulfonamide containing antibiotic ACM/DACM and lay a solid foundation for target improvement of their production via synthetic biology strategy..

## Results

### Identification and characterization of the ascamycin/dealanyl-ascamycin biosynthetic genes

**Figure 2:**
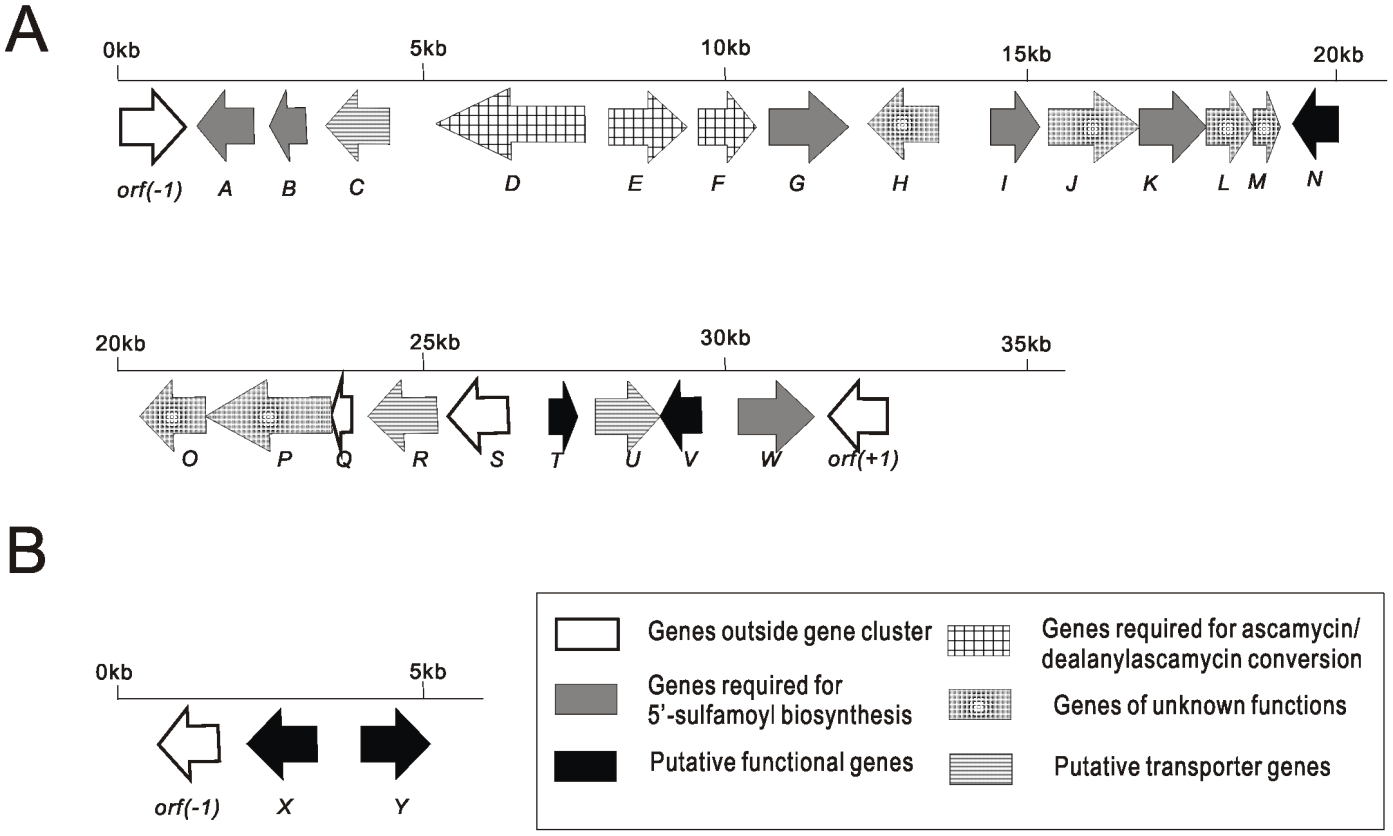
Gene organization of ascamycin/dealanylascamycin biosynthesis pathway. A) AcmA to AcmW. B) Chlorinases *acmX* and *acmY*.

*Streptomyces sp.* JCM9888 is unusual in its ability to biosynthesize two nucleoside antibiotics: dealanylascamycin (**1**) and ascamycin (**2**) [20] (Fig. 1). As they are both featured with a 5′-O-sulfonamide moiety, we suspected sulfate metabolite related genes are required for the antibiotic production. Partial genome sequencing of *Streptomyces* genome revealed a 30,488 bp contiguous DNA sequence with an overall GC content of 66.7% (GenBank accession number KJ817374). Bioinformatics analysis of the sequence revealed 23 ORFs (*acmA-W*) and the deduced functions of individual ORFs are annotated and summarized in Table 1. Of them, 6 genes are proposed to be related to 5′-O-sulfonamide biosynthesis (*acmA*, *acmB*, *acmG*, *acmI*, *acmK*, *acmW*) (Fig. 2A). Notably, no halogenases were obvious around the gene cluster while BLASTp searches highlighted that two hypothetical flavin-dependent chlorinases (*acmX* and *acmY*) (GenBank accession number KJ817375) lying adjacent to each other on the chromosome in a distance of approx. 1 million basepair from *acmA-W* (Fig. 2B). AcmX and AcmY were shown to share high homology to all known FAD-dependent chlorinases, such as ChlB4 (accession number AAZ77674) from *Streptomyces antibiotics* which participate in chlorothricin biosynthesis (65% and 57% protein sequence identity respectively) [32]. Their analogy to proteins required for aromatic moiety chlorination suggested that they are potential candidates of the ascamycin/dealanylascamycin chlorinases as both two antibiotics possess a chlorine atom at C2-position of adenine.

**Table 1:** Deduced ORFs and their predicted functions in the ascamycin/dealanylascamycin gene cluster.

Gene	Product size	Purposed function	Closest relative, origin, accession number	%identity
*orf(-1)*	350	transposase	*Orf3*, *Streptomyces rishiriensis,* AAR11882	69
*acmA*	320	Sulfate adenylyltransferase subunit 2	*MmcV*, *Streptomyces lavendulae*, Q9X5U0	80
*acmB*	179	adenylylsulfate kinase	OrfQ, *Streptomyces* sp. AM-7161, BAC79014	67
*acmC*	414	major facilitator superfamily protein	*Micromonospora* sp. CNB394, WP_018786560	29
*acmD*	871	alanyl-tRNA synthetase	M901_2280, *Bacteriovorax* sp. DB6_IX, EQC50866	44
*acmE*	455	esterase	*Xanthomonas campestris*, BAA11623	20
*acmF*	370	alanyl-tRNA synthetase	CGL2_10933004, *Leptospirillum* sp. Group II '5-way CG', EDZ38785	27
*acmG*	480	Sulfatase	Francci3_1756, *Frankia* sp. CcI3, YP_480861	47
*acmH*	453	Radical SAM domain/B12 binding domain-containing protein	MCON_2786, *Methanosaeta concilii*, YP_004384990	24
*acmI*	302	acylsulfatase	*Streptomyces* sp. CNB091, WP_018955485	69
*acmJ*	557	Putative Fe-S oxidoreductase	*Streptomyces clavuligerus*, WP_003962555	79
*acmK*	356	sulfotransferase	SGR_905, *Streptomyces griseus*, YP_001822417	59
*acmL*	250	methyltransferase	UbiE, *Leifsonia rubra* CMS 76R, EPR77097	30
*acmM*	142	hypothetical protein	BN159_7117, *Streptomyces davawensis*, YP_007525623	77
*acmN*	319	amidinotransferase	Francci3_1759, *Frankia* sp. CcI3, YP_480864	57
*acmO*	444	hypothetical protein	Francci3_1760, *Frankia* sp. CcI3, YP_480865	54
*acmP*	658	DNA topoisomerase II	Francci3_1761, *Frankia* sp. CcI3, YP_480866	68
*acmQ*	155	rubrerythrin	Francci3_1762, *Frankia* sp. CcI3, YP_480866	54
*acmR*	438	major facilitator superfamily protein	*Streptomyces* sp. W007, WP_007453428	53
*acmS*	395	hypothetical proteins	*Streptomyces griseus*, WP_003963977	31
*acmT*	220	phosphatase	*Streptomyces sulphureus*, WP_019549800	52
*acmU*	418	sodium/hydrogen antiporter	*flH*, *Streptomyces cattleya*, CAJ20009	28
*acmV*	194	adenine phosphoribosyl-transferase	*Streptomyces albulus*, WP_016574607	58
*acmW*	451	sulfate adenylyltransferase large subunit	Azi10, *Streptomyces sahachiroi*, ABY83149	78
*orf(+1)*	323	oxidoreductase	*Streptomyces scabrisporus*, WP_020552419	85
*acmX*	449	flavin-dependent halogenase	ChlB4, *Streptomyces antibioticus*, AAZ77674	65
*acmY*	438	flavin-dependent halogenase	ChlB4, *Streptomyces antibioticus*, AAZ77674	57

### 5′-O-sulfonamide and 5′-(*N*-alanyl-O-sulfonamide) groups formation

**Figure 3:**
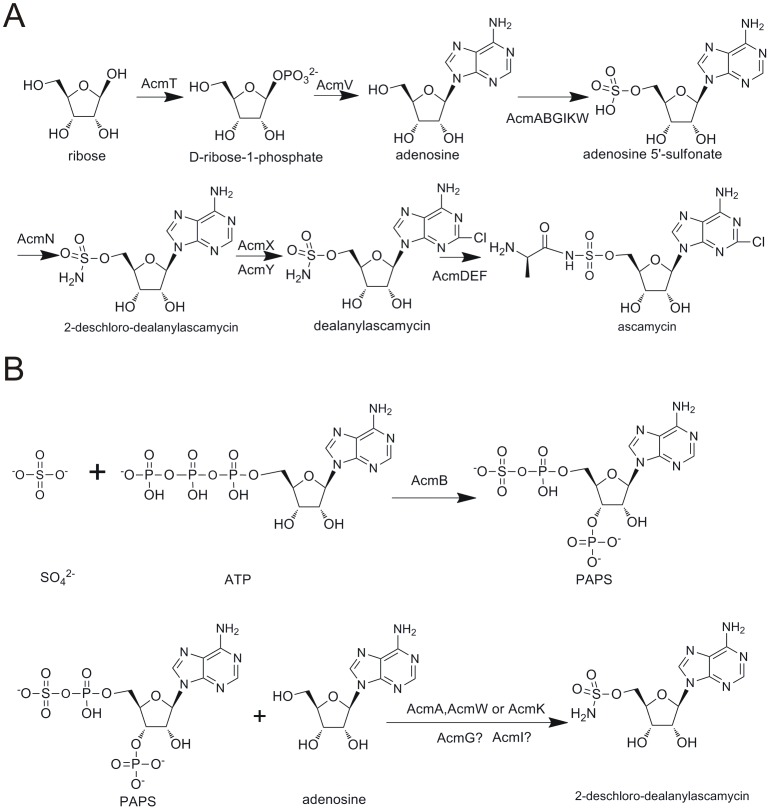
Proposed model for Acm/Dacm and 5′-O-sulfonamide group biosynthesis. A) Biosynthetic pathway of ascamycin/dealanylascamycin. B) Biosynthetic pathway for 5′-O-sulfonamide group formation.

Ascamycin and dealanylascamycin are decorated with unusual sulfonamide groups at the 5′-hydroxyl of the ribose ring which are rare paradigms in secondary metabolites [33]. Six genes are postulated to be involved in 5′-O-sulfonamide moiety activation, loading and transfer in the ascamycin/dealanylascamycin gene cluster (*acmA*, *B*, *G*, *I*, *K*, *W*). Sulfate ions(SO_4_^2−^) are not utilised directly but must first be activated by the formation of adenosyl phosphosulfate (APS) or phosphoadenosine phosphosulfate (PAPS). These are activated forms of sulfate generated by coupling inorganic sulfate onto ATP (adenosine triphosphate) (Fig. 3B). Sulfurylation of ATP is probably catalysed by adenylsulfate kinase (AcmB): SO_4_^2−^ + ATP → PAPS + PP_i_. Next, AcmK (sulfotransferase) [34] or AcmA and AcmW (sulfate adenyltransferase) [35] act upon the 5′ position of adenosine, utilizing PAPS as an activated sulfate donor to generate adenosine 5′-sulfonate. The manner in which AcmG (sulfatase) and AcmI (acylsulfatase) participate in this complex sulfate metabolite pathway is still elusive although bioinformatics analyses suggest that they have relevant functions (Fig. 3B). An aminotransferase AcmN was postulated to generate the 5′-O-sulfonamide moiety by replacing a hydroxyl group of the sulfonate with an amine group (Fig. 3B).

### Verification of the ascamycin/dealanylascamycin biosynthetic genes

**Figure 4:**
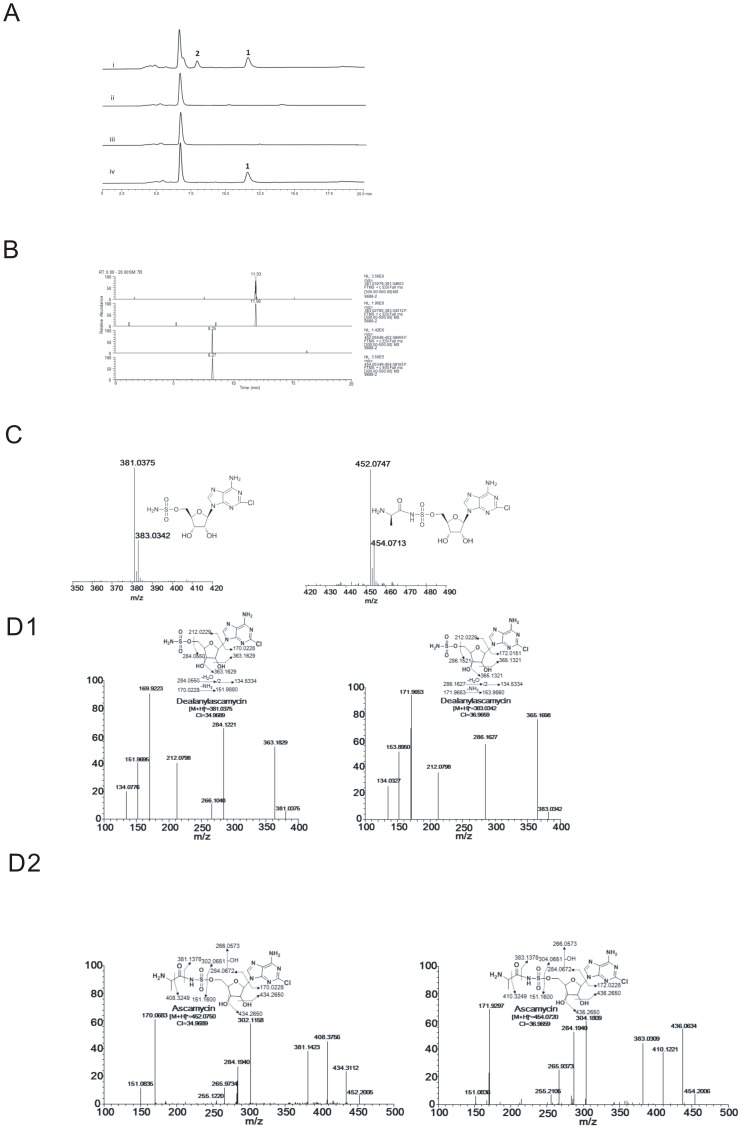
Analysis of metabolite profiles of *Streptomyces* JCM9888 and related mutants. A) HPLC-UV analysis of metabolite profiles of *Streptomyces*: i) Wild type JCM9888, ii) Δ*acmG* mutant, iii) Δ*acmK* mutant, iv) Δ*acmE* mutant. The products DACM (**1**), ACM (**2**) are indicated. B) HPLC-ESI-MS analysis of metabolite profile of JCM9888 under the same program of HPLC-UV. C) The molecular formula of compounds DACM (**1**), ACM (**2**) were determined to be C_10_H_13_ClN_6_O_6_S (*m*/*z*  = 381.0384, 383.0354 [M+H]^+^) and C_13_H_18_ClN_7_O_7_S (*m*/*z*  = 452.0755, 454.0725 [M+H]^+^) on the basis of ESI-HRMS. D) MS/MS pattern of ascamycin and dealanylascamycin are shown and the detected data is closely consistent with anticipated fragmentation ions. D1: dealanylascamycin; D2: ascamycin.

To verify our hypothesis that this sulfate related pathway is responsible for ascamycin/dealanylascamycin biosynthesis, a genomic cosmid library of JCM9888 was constructed on shuttle vector SuperCos1. Using *acmG* gene as a DNA probe, four positive clones were identified from some 4,000 packaged cosmids. Gene inactivation was carried out to disrupt *acmG* (sulfatase) or *acmK* (sulfotransferase) respectively (See material and methods) and the resulted mutants were validated by PCR analysis and subsequent sequencing using primers listed in Table S2 (See results in Fig. S1). When the *Streptomyces* mutants were fermented on petri dishes, as anticipated they lose their ability to elaborate both ascamycin and dealanylascamycin, as showed in HPLC-UV analysis (Fig. 4A). The molecular formula of compounds 1 and 2 were determined to be C_10_H_13_ClN_6_O_6_S (*m*/*z*  =  381.0384, 383.0354 [M+H]^+^) and C_13_H_18_ClN_7_O_7_S (*m*/*z*  = 452.0755, 454.0725 [M+H]^+^) on the basis of ESI-HRMS, the isotopic pattern indicated ascamycin and dealanylascamycin each contained one Cl atom (Fig. 4BC). Detailed MS/MS pattern of ascamycin and dealanylascamycin are shown (Fig. 4D). The detected data is closely consistent with anticipated fragmentation ions and every mass of debris have been elucidated (Fig. 4D1: dealanylascamycin; Fig. 4D2: ascamycin).

### AcmE is an esterase which is responsible for the conversion of dealanylascamycin to ascamycin

Analysing ascamycin/dealanylascamycin gene cluster also revealed two alanyl-tRNA synthetases (*acmD* and *acmF*). Intriguingly, ascamycin has an alanyl-cap while dealanylascamycin lacks it. Gene *acmE* located between *acmD* and *acmF* whose translated product weakly resemble an aminopeptidase from *Xanthomonas campestris* (accession number BAA11623, 20% identity) which have been demonstrated *in vitro* to catalyse a hydrolysis reaction of ascamycin to dealanylascamycin causing the host to become susceptible to ascamycin [36]. Most bacteria lack this enzyme and therefore are resistant to ascamycin and this explain the very different bacterial activity spectrum of ascamycin and dealanylascamycin [37]. The location of *acmCDE* in the biosynthetic pathway suggests their role in converting dealanylascamycin to ascamycin *via* a tRNA-dependent manner, in which *acmE* may catalyse a condensation reaction between alanyl-tRNA and dealanylascamycin to generate ascamycin [38].

To verify above hypothesis, gene inactivation was carried out on *acmE* and the *Streptomyces* mutant was validated by PCR on appropriate primers (Fig. S1-A). Intriguingly, *acmE*-null mutant could only produce dealanylascamycin but was blocked in its ability to biosynthesize ascamycin, as illustrated in HPLC-UV analysis (Fig. 4A(iv)), confirming the role of *acmE* to catalyse the condensation reaction of dealanylascamycin and L-alanine to generate ascamycin. (Fig. 3A)

### A proposed model for ACM/DACM biosynthesis

Thus we propose a model for ascamycin/dealanylascamycin biosynthesis as illustrated in Fig. 3A. Firstly, a phosphatase (AcmT) mediates phosphorylation at C1 of D-ribose to generate D-ribose-1-phosphate [39]. The next enzyme, a phosphoribosyltransferase (AcmV) catalyze the displacement of the phosphate group by adenine to form adenosine [40]. 5′-O-sulfonamide biosynthesis related enzymes (AcmABGIKW) activate inorganic sulfate anions and install a sulfonate group onto the 5′ position of adenosine. Then AcmN, an aminotransferase transfers an amine group onto the sulfonate moiety to generate the corresponding O-sulfonamide, which is dealanylascamycin. Finally, AcmE catalyse an amide formation between the carboxyl end of L-alanine and the NH_2_ on the O-sulfonamide group of dealanylascamycin in a tRNA-dependent manner to furnish ascamycin [38].

## Discussion

Nucleoside antibiotics are a large family of compounds with significant activities and some of them have been developed into antibacterial, antifungal and anti-parasite agents. The pace of discovering their biosynthetic pathways were increasing in the past decade, revealing new enzymes and paving the way to new “unnatural” natural products by synthetic biology. Ascamycin/dealanylascamycin are unusual nucleoside antibiotics which are elaborated by *Streptomyces sp*. They share a special 5′-O-sulfonamide moiety on adenosine which is not present in other antibiotics to the best of our knowledge. In this study we identified the putative genes which are involved in their biosynthetic pathway, including those genes required for sulfonamide formation, chlorination and *N*-alanylation. The details of how dealanylascamycin is converted into ascamycin is an intriguing question that has yet to be answered. Here we present *in vivo* evidence to prove our hypothesis that an esterase AcmE are responsible for that. Bioinformatics analysis showed that an esterase AcmE was present which also had some similarity to the only aminopeptidase identified to-date that cleaves ascamycin to generate dealanylascamycin. The host only becomes (*Xanthomonas sp.*) susceptibile to ascamycin [24] when they have this aminopeptidase activity. We suspect *acmE* is involved in a reversible *N*-alanylation enzymatic reaction in the ascamycin/dealanylascamycin pathway.

Ascamycin and dealanylascamycin are rare secondary metabolites decorated with sulfonamide groups at the 5′-hydroxyl of the ribose ring. Six organosulfur metabolism genes (AcmABGKIW) and aminotransferase AcmN are candidate genes required for 5′-O-sulfonamide formation. These genes showed little homology to other genes of sulfur-groups metabolism [eg. The caprazamycin metabolite pathway which involves a two-step sulfation:A triketide pyrones that are sulfated by an PAPS-dependent sulfotransferase (Cpz8) to generate phenolic sulfate esters which play a role as sulfate donors for a PAPS-independent arylsulfate sulfotransferase (Cpz4) to produce sulfated licaprazamycin(s) [41]]. Another intriguing question is halogenation of ACM/DACM, which probably involved AcmX and/or AcmY. Though they are 1Mb remote from AcmA-AcmW gene cluster, they can be regarded as candidate for adenine C-2 halogenase because they showed considerable homology to all FAD-type halogenase which are believed to be required for aromatic adenine ring chlorination. When JCM9888 genome was aligned with non-heme iron halogenases, SAM-dependent halogenases or vanadium and heme-dependent halogenses, no counterpart genes or homologues were identified [25] [26] [27]. We also checked the mycelium and spore formation of *Streptomyces* mutant Δ*acmE*, Δ*acmG*, Δ*acmK* but did not observed any difference between them and wild type JCM9888, suggesting that these genes or secondary metabolites ACM/DACM are not absolutely required for morphological differentiation.

It is an important aim of our project to provide more information to investigate the enigmatic fluorinated natural compound nucleocidin, which was discovered in 1969 [3], while all efforts to identify this famous fluorinated natural products from *Streptomyces* deposited in public collections failed in the past twenty years (Prof. David O′Hagan, University of St Andrews, UK, personal communication). Nucleocidin possess a very similar chemical structure with dealanylascamycin except that nucleocidin has a fluorine atom at C4′ of the ribose while the later is chlorinated at C2 position of the adenine ring. We suspect that these two metabolic pathways use a set of very similar genes and proteins. Given access to the recently published genome of the putative nucleocidin producer *Streptomyces calvus* DSM40010 [42], we aligned the ascamycin/dealanylascamycin gene cluster against the *S. calvus* genome database, but no homologous counterpart was identified, suggesting that commercially obtained cultures of *S. calvus* DSM40010 (or ATCC13382) may not be the correct nucleocidin-producing strain. We suspect that a true nucleocidin-producing *Streptomyces* would also carry the related genes of ACM/DACM biosynthesis. Thus we envisage that with certain effort, it would be possible to re-isolate a nucleocidin-producing *Streptomyces* strain from such colonies by soil screening approach. Knowledge of the genes of related ACM/DACM biosynthesis would be necessary in order to devise PCR screening primers. This target-narrowing using gene PCR provides obvious advantages over verification by fermentation, extraction and metabolite analyses of candidate *Streptomyces* colonies individually.

## Methods

### Bacterial strains, plasmids and reagents

Bacterial strains and plasmids used and constructed in this study are listed in Table S1. Primers were chemically synthesized by commercial resources and listed in Table S2. strains JCM9888 were grown at 28°C on ISP4 medium for growth and sporulation [43]. MS medium (soy bean flour 20 g, mannitol 20 g, agar 20 g, 2.5 mM MgSO_4_, 2.5 mM KCl in 1 L of tap water, pH 8.0) is used for petri dish fermentation of antibiotics ascamycin/dealanylascamycin [20], [21]. ISP4 medium supplemented with 0.2% yeast extract is used for *E. coli-Streptomyces* bi-parental conjugation.

### DNA extraction, manipulation and sequencing

Total DNA isolations, plasmid DNA preparations, restriction endonuclease digestions, ligations, and other DNA manipulations were performed according to standard procedures for *E. coli* and *Streptomyces* [43] [44]. The sequencing service at the Chinese National Genome Centre (Shanghai) was used for DNA sequencing. Computer-assisted database searching and sequence analysis were carried out online using the frame-plot 4.0 sequence analysis software (http://nocardia.nih.go.jp/fp4/) and the BLAST program (http://blast.ncbi.nlm.nih.gov/Blast.cgi).

### Gene Inactivation Experiments

The lambda-RED-mediated gene replacements were performed as standard procedures [45]. In all cases, the *aac(3)IV* gene from pIJ773 was inserted within the coding region of the gene to be disrupted and in the same direction of transcription. The introduction of DNA into JCM9888 was achieved through intergeneric conjugation from *E. coli* ET12567/pUZ8002. After the introduction of the constructs into *Streptomyces* by intergeneric conjugation, exconjugants, in which a double crossover occurred, were identified by their resistance to apramycin and susceptibility to kanamycin.

To generate a mutant containing a deletion or an insertion mutation, the following primers were designed: (i) Mutagenesis of AcmE (esterase): AcmE-FP: CCC GGC CAA CCG CGC TTC ATC TGC TCG ACG TTG ACC GTC att ccg ggg atc cgt cga cc, AcmE-RP: CCG CAA CGA CCC GCG TGC GCC GCC CCG ATG GTC AAG CGG tgt agg ctg gag ctg ctt c. (ii) Mutagenesis of AcmG (Sulfatase): AcmG-FP: GAG GGG AGA CGC TCT TCA CCT GGG CGC GCC GAC GCG GCT att ccg ggg atc cgt cga cc, AcmG-RP: CGA GGA TGA TCG GGA AGT CGC TCT TGA AGT ACG TCC GCA tgt agg ctg gag ctg ctt c. (iii) Mutagenesis of AcmK (sulfotransferase): AcmK-FP: GTC CGA GAC CGC ACG TCG CGC CGC GAC CCG GGA CGC CCT att ccg ggg atc cgt cga cc, AcmK-RP: TCA CAG CCC CAC GGG TGG ATG TGC GTG CGC AGG AGG GCC tgt agg ctg gag ctg ctt c.

The apramycin resistance cassette pIJ773 was used as a template throughout the experiments. The PCR products were transferred into competent cells of BW25113 (pIJ790, cosmid 15H6) to isolate apramycin-resistant and amplicillin-resistant *E. coli* colonies. These mutated cosmids were introduced into ET12567 (pUZ8002) and then transferred by conjugation to JCM9888 to screen for apramycin resistant and kanamycin-sensitive colonies. This resulted in Δ*acmE*, Δ*acmG* and Δ*acmK* mutants respectively, which were validated by PCR and subsequent sequencing. (Fig. S1)

### Production, purification and analysis of ascamycin/dealanylascamycin

Ascamycin/dealanylascamycin isolation were performed according to the literature [20] [46]. Fermentation agar from petri dishes (weighing approx. 200 g) was extracted with 60% aqueous acetone. After removal of acetone by rotatory evaporation, the residual extract was passed through a Dowex 80WX8(H) resin column (20 ml), which was then eluted by 0.5 M ammonia solution.

The HPLC-MS analysis of the antibiotics obtained from above was performed on a C-18 reversed-phase column (150 by 4.6 mm; particle size, 5 mm [Phenomenex]) with UV detection at 258 nm. The solvent system consisted of solvent A (0.1% formic acid in water), and solvent B (CH_3_CN). The HPLC gradient was as follows: 10 % to 55 % solvent B (0 to 15 min), 55 % solvent B (15 to 18 min), 55 % to 10 % solvent B (18 to 20 min), and 10 % solvent B (20 to 25 min) at a flow rate of 0.5 ml/min.

#### Nucleotide sequence accession number

The complete sequence of the ascamycin/dealanylascamycin biosynthetic gene cluster and annotated proteins have been deposited in GenBank under accession number (GenBank accession number KJ817374 and KJ817375).

## Supporting Information

Figure S1 **PCR validation of** ***Streptomyces*** **mutants.**
(TIF)

Table S1 **Strains, plasmids and cosmids used in this study.**
(DOCX)

Table S2 **PCR primers used in this study.**
(DOCX)

Data S1 **Supplementary data associated with this article.**
(DOCX)
