# Supplementary figures and images for: Characterization of Biosynthetic Genes of Ascamycin/Dealanylascamycin Featuring a 5′-O-Sulfonamide Moiety in Streptomyces sp. JCM9888
Source: PLoS One. 2014 Dec 5;9(12):e114722. doi: 10.1371/journal.pone.0114722 (PMC4257720; doi:10.1371/journal.pone.0114722)

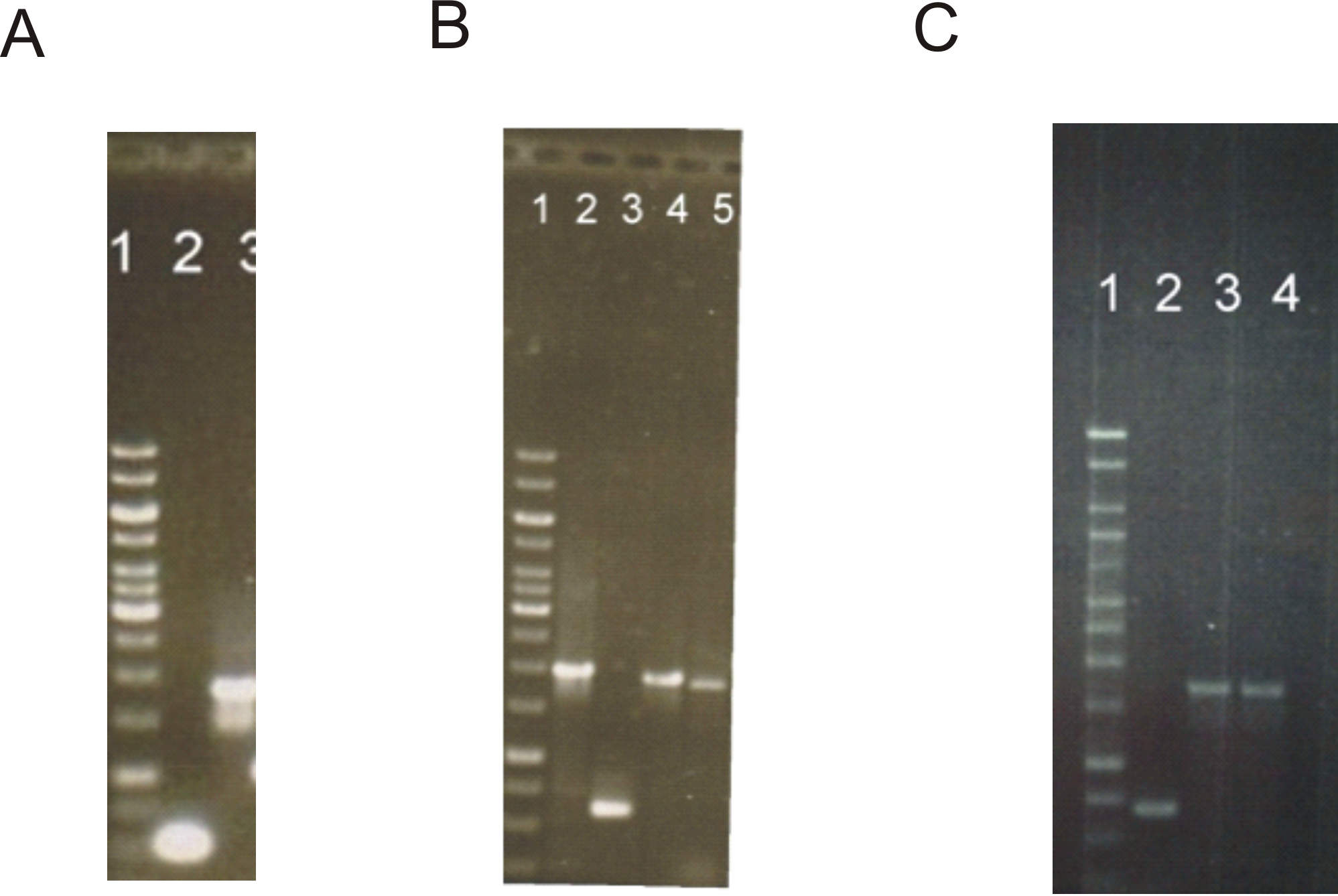

Supplement: Figure S1 — PCR validation of Streptomyces mutants. (TIF) [file pone.0114722.s001.tif]
