# Supplementary material for: Characterization of Biosynthetic Genes of Ascamycin/Dealanylascamycin Featuring a 5′-O-Sulfonamide Moiety in Streptomyces sp. JCM9888
Source: PLoS One. 2014 Dec 5;9(12):e114722. doi: 10.1371/journal.pone.0114722 (PMC4257720; doi:10.1371/journal.pone.0114722)
Supplement: Table S1 — Strains, plasmids and cosmids used in this study. (DOCX) [file pone.0114722.s002.docx]

**Table S1. Strains, plasmids and cosmids used in this study**

| **Strain/Plasmid /Cosmid** | **Relevant characteristics*** | **Reference or source** |
| --- | --- | --- |
| ***Streptomyces* JCM9888 strains** |  |  |
| JCM9888 | Wild-type of ascamycin/ dealanylascamycin producing strain | 1 |
| CZ1 | Dealanylascamycin producer generated through disruption of *acmE* by *aac(3)IV* | This study |
| CZ2 | Non-producer for Acm/Dacm generated through disruption of *acmG* by *aac(3)IV* | This study |
| CZ3 | Non-producer for ACM/DACM generated through disruption of *acmK* by *aac(3)IV* | This study |
| ***E. coli* strains** |  |  |
| DH10B | Cloning host | GIBCO BRL |
| ET12567 (pUZ8002) | *E. coli*-*Streptomyces* conjugation | 2 |
| **Plasmids** |  |  |
| pZC1001 | Construct for *acmE* inactivation | This study |
| pZC1002 | Construct for *acmG* inactivation | This study |
| pZC1003 | Construct for *acmK* inactivation | This study |
