# Supplementary material for: Characterization of Biosynthetic Genes of Ascamycin/Dealanylascamycin Featuring a 5′-O-Sulfonamide Moiety in Streptomyces sp. JCM9888
Source: PLoS One. 2014 Dec 5;9(12):e114722. doi: 10.1371/journal.pone.0114722 (PMC4257720; doi:10.1371/journal.pone.0114722)
Supplement: Table S2 — PCR primers used in this study. (DOCX) [file pone.0114722.s003.docx]

**Table S2. PCR primers used in this study**

| Primers | Sequence |
| --- | --- |
| AcmE-FP | CCC GGC CAA CCG CGC TTC ATC TGC TCG ACG TTG ACC GTC att ccg ggg atc cgt cga cc |
| AcmE-RP | CCG CAA CGA CCC GCG TGC GCC GCC CCG ATG GTC AAG CGG tgt agg ctg gag ctg ctt c |
| AcmG-FP | GAG GGG AGA CGC TCT TCA CCT GGG CGC GCC GAC GCG GCT att ccg ggg atc cgt cga cc |
| AcmG-RP | CGA GGA TGA TCG GGA AGT CGC TCT TGA AGT ACG TCC GCA tgt agg ctg gag ctg ctt c |
| AcmK-FP | GTC CGA GAC CGC ACG TCG CGC CGC GAC CCG GGA CGC CCT att ccg ggg atc cgt cga cc |
| AcmK-RP | TCA CAG CCC CAC GGG TGG ATG TGC GTG CGC AGG AGG GCC tgt agg ctg gag ctg ctt |
| AcmE-FP2 | GTCTTGAGTGCGCTCGTTGC |
| AcmE-RP2 | GAGGCTCTGTTCGGTGGTGT |
| AcmG-FP2 | CAAACCCGTTGAAGCTCTGG |
| AcmG-RP2 | CGTAGTCGCCGCTTTCGTAG |
| AcmK-FP2 | CTCAACGCCCACCCGTCCAT |
| AcmK-RP2 | TCCGGTCAAGTGCGGAGCAA |
