# Supplementary material for: Characterization of Biosynthetic Genes of Ascamycin/Dealanylascamycin Featuring a 5′-O-Sulfonamide Moiety in Streptomyces sp. JCM9888
Source: PLoS One. 2014 Dec 5;9(12):e114722. doi: 10.1371/journal.pone.0114722 (PMC4257720; doi:10.1371/journal.pone.0114722)
Supplement: Data S1 — Supplementary data associated with this article. (DOCX) [file pone.0114722.s004.docx]

**Supplemental Information**

**Characterization of Biosynthetic Genes of Ascamycin/ Dealanylascamycin Featuring a 5’-O-sulfonamide Moiety in *Streptomyces sp.* JCM9888**

Chunhua Zhao^1^*, Jianzhao Qi^1^, Weixing Tao^1^, Lei He^1^, Wei Xu^1^, Jason Chan^2^, Zixin Deng^1^*

^1^Key Laboratory of Combinatory Biosynthesis and Drug Discovery (Ministry of Education) and School of Pharmaceutical Sciences, Wuhan University, 185 East Lake Road, Wuhan 430071, PR China. ^2^Department of Chemistry, The Hong Kong University of Science and Technology, Clear Water Bay, Kowloon, Hong Kong. *For correspondence: Chunhua Zhao: Tel: (0086)-27-68755235, Email: zhaochunhua@whu.edu.cn or Zixin Deng: Tel: (0086)-27-62933404, Email: [zxdeng@sjtu.edu.cn](mailto:zxdeng@sjtu.edu.cn)

**Inventory**

**Table S1. Strains, plasmids and cosmids used in this study**

**Table S2. PCR primers used in this study**

**Figure S1. PCR validation of *Streptomyces* mutants.**

**Supplemental References**

**Table S1. Strains, plasmids and cosmids used in this study**

| **Strain/Plasmid /Cosmid** | **Relevant characteristics*** | **Reference or source** |
| --- | --- | --- |
| ***Streptomyces* JCM9888 strains** |  |  |
| JCM9888 | Wild-type of ascamycin/ dealanylascamycin producing strain | 1 |
| CZ1 | Dealanylascamycin producer generated through disruption of *acmE* by *aac(3)IV* | This study |
| CZ2 | Non-producer for Acm/Dacm generated through disruption of *acmG* by *aac(3)IV* | This study |
| CZ3 | Non-producer for ACM/DACM generated through disruption of *acmK* by *aac(3)IV* | This study |
| ***E. coli* strains** |  |  |
| DH10B | Cloning host | GIBCO BRL |
| ET12567 (pUZ8002) | *E. coli*-*Streptomyces* conjugation | 2 |
| **Plasmids** |  |  |
| pZC1001 | Construct for *acmE* inactivation | This study |
| pZC1002 | Construct for *acmG* inactivation | This study |
| pZC1003 | Construct for *acmK* inactivation | This study |

**Table S2. PCR primers used in this study**

| Primers | Sequence |
| --- | --- |
| AcmE-FP | CCC GGC CAA CCG CGC TTC ATC TGC TCG ACG TTG ACC GTC att ccg ggg atc cgt cga cc |
| AcmE-RP | CCG CAA CGA CCC GCG TGC GCC GCC CCG ATG GTC AAG CGG tgt agg ctg gag ctg ctt c |
| AcmG-FP | GAG GGG AGA CGC TCT TCA CCT GGG CGC GCC GAC GCG GCT att ccg ggg atc cgt cga cc |
| AcmG-RP | CGA GGA TGA TCG GGA AGT CGC TCT TGA AGT ACG TCC GCA tgt agg ctg gag ctg ctt c |
| AcmK-FP | GTC CGA GAC CGC ACG TCG CGC CGC GAC CCG GGA CGC CCT att ccg ggg atc cgt cga cc |
| AcmK-RP | TCA CAG CCC CAC GGG TGG ATG TGC GTG CGC AGG AGG GCC tgt agg ctg gag ctg ctt |
| AcmE-FP2 | GTCTTGAGTGCGCTCGTTGC |
| AcmE-RP2 | GAGGCTCTGTTCGGTGGTGT |
| AcmG-FP2 | CAAACCCGTTGAAGCTCTGG |
| AcmG-RP2 | CGTAGTCGCCGCTTTCGTAG |
| AcmK-FP2 | CTCAACGCCCACCCGTCCAT |
| AcmK-RP2 | TCCGGTCAAGTGCGGAGCAA |

Figure S1. PCR validation of *Streptomyces* mutants. A) PCR validation of *AcmE*-null mutants. Lane 1: 1kb ladder. Lane2: genomic DNA of wild type *S. sp.* JCM9888. PCR by primers *AcmE* -FP2 and *AcmE* -RP2 (0.5kb band). Lane3: genomic DNA of *AcmE*-null mutant PCR by primers *AcmE* -FP2 and *AcmE* -RP2 (1.8kb band). B) PCR validation of *AcmG*-null mutants. Lane 1: 1kb ladder. Lane2, 4, 5: genomic DNA of *AcmG-*null mutant PCR by primers AcmG-FP2 and AcmG-RP2 (2.0kb band). Lane3: genomic DNA of wild type *S. sp. JCM9888.* PCR by primers *AcmG* -FP2 and *AcmG* -RP2 (0.7kb band). C) PCR validation of *AcmK*-null mutants. Lane 1: 1kb ladder. Lane2: genomic DNA of wild type *S. sp. JCM9888* PCR by primers *AcmK* -FP2 and *AcmK*-RP2 (0.9kb band). Lane3-4: genomic DNA of *AcmK*-null mutant PCR by primers *AcmK* -FP2 and *AcmK* -RP2 (2.2kb band).

**
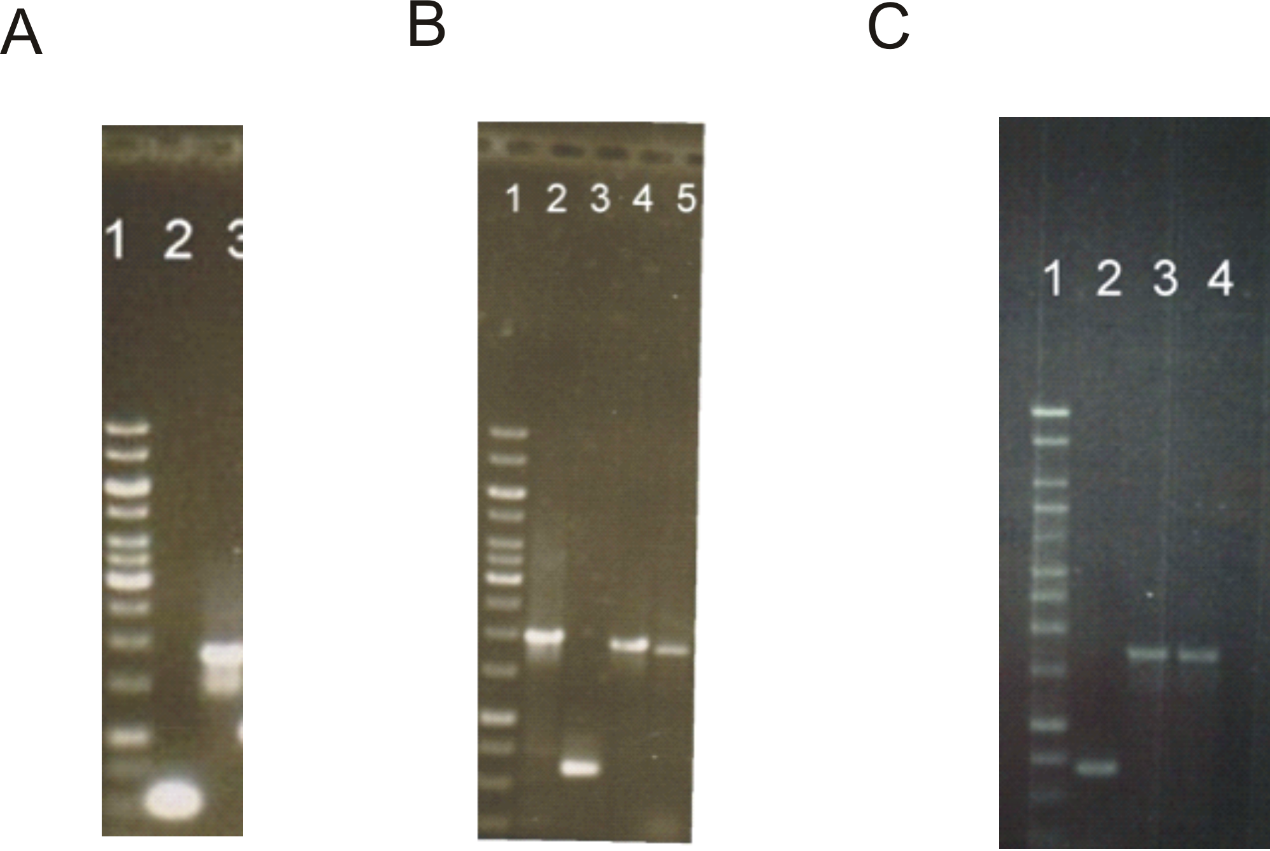
**

**Supplemental References**

1. Isono K, Uramoto M, Kusakabe H, Miyata N, Koyama T, et al. (1984) Ascamycin and dealanylascamycin, nucleoside antibiotics from Streptomyces sp. J Antibiot (Tokyo) 37: 670-672.

2. Kieser T, Bibb M, Butter M, Chater KF, Hopwood DA (2001) Practical Streptomyces Genetics. The John Innes Foundation, Norwich, United Kingdom.
